# The Importance of Encoding-Related Neural Dynamics in the Prediction of Inter-Individual Differences in Verbal Working Memory Performance

**DOI:** 10.1371/journal.pone.0069278

**Published:** 2013-07-09

**Authors:** Steve Majerus, Eric Salmon, Lucie Attout

**Affiliations:** 1 Department of Psychology - Cognition & Behaviour, Université de Liège, Liège, Belgium; 2 Cyclotron Research Centre, Université de Liège, Liège, Belgium; 3 Fund for Scientific Research FNRS, Brussels, Belgium; Emory University, United States of America

## Abstract

Studies of brain-behaviour interactions in the field of working memory (WM) have associated WM success with activation of a fronto-parietal network during the maintenance stage, and this mainly for visuo-spatial WM. Using an inter-individual differences approach, we demonstrate here the equal importance of neural dynamics during the encoding stage, and this in the context of verbal WM tasks which are characterized by encoding phases of long duration and sustained attentional demands. Participants encoded and maintained 5-word lists, half of them containing an unexpected word intended to disturb WM encoding and associated task-related attention processes. We observed that inter-individual differences in WM performance for lists containing disturbing stimuli were related to activation levels in a region previously associated with task-related attentional processing, the left intraparietal sulcus (IPS), and this during stimulus encoding but not maintenance; functional connectivity strength between the left IPS and lateral prefrontal cortex (PFC) further predicted WM performance. This study highlights the critical role, during WM encoding, of neural substrates involved in task-related attentional processes for predicting inter-individual differences in verbal WM performance, and, more generally, provides support for attention-based models of WM.

## Introduction

While completing working memory (WM) tasks, a fronto-parietal network, composed of the anterior and posterior intraparietal sulci (IPS) and the lateral prefrontal cortex is consistently activated and has been considered to reflect the core neural substrate of WM [1], [2]. At the same time, the relationship between the activation of this network during WM tasks and actual behavioural success on these tasks is less well understood. Given the well-documented, large inter-individual differences that characterize WM performance, with typical digit list repetition spans ranging between 5 and nearly twice as much in young adults [3], it is critical to understand how these inter-individual differences in behaviour relate to inter-individual variations in the functional network and underlying cognitive processes that support WM. Group-based activation studies inform us about the functional neural architecture that is common across a group of individuals for a given cognitive task but they do not inform us about the variability of this neural architecture and how it explains inter-individual differences in cognitive performance. The aim of this study is to further our understanding of brain-behaviour interactions during WM, with a specific focus on verbal WM where individual differences in behavioural performance are particularly large [3].

On the one hand, our understanding of the functional neural architecture activated during the completion of WM tasks is getting more and more precise. Many studies now agree on the important role of the intraparietal cortex in WM tasks, by showing that the bilateral IPS, in both anterior and posterior parts, is sensitive to memory load during short-term retention tasks, and this for both verbal and visual WM tasks [4], [5], [6]. The same has also been shown for the bilateral dorsolateral prefrontal cortex [6], [7]. Furthermore, activation dynamics in the intraparietal cortex parallel WM capacity limitations : activation in the IPS has been shown to reach a plateau at about 4–6 items to be maintained in WM, corresponding to the well-known behavioural capacity limits of 4–6 items in visual WM [4], [8], [9]. More generally, an increasing number of studies associate the fronto-parietal network involved in WM with attention networks, by demonstrating that this network includes the dorsal attention stream which allows attention to be directed in a task-related manner upon the stimuli to be encoded and to be maintained [1,4,6 9]. Especially the parietal and the superior frontal subparts of this network have been shown to be involved in task-related attentional selection processes [4], [10]. With respect to the prefrontal cortex, the dorsolateral prefrontal cortex during WM tasks has been associated with executive control and monitoring processes, and more precisely resistance to proactive interference, while the ventrolateral prefrontal cortex is associated with articulatory rehearsal processes especially in the domain of verbal WM although some authors also associate this region with proactive interference resolution [11], [12], [13].

However, despite the increasing precision of our knowledge concerning the functional neural architecture activated during WM tasks, the relationship between this architecture and the variability of WM performance remains poorly understood. Some studies exploring brain-behaviour interactions in WM tasks used an intra-individual approach, by differentiating correct from incorrect trials, and by distinguishing brain activity for correct and incorrect trials in each individual [14], [15], [16]. These studies have led to controversial findings: a study by Pessoa et al. showed higher activation in dorsolateral prefrontal cortex and posterior parietal cortex for correct *versus* incorrect trials; another study by Satterhwaite et al. [16] showed higher activation in the bilateral dorsolateral prefrontal cortex for incorrect *versus* correct trials; finally, Anticevic et al. reported increased deactivation in the temporo-parietal junction for correct trials. These discrepant results are likely to be related to the different types of paradigms used (delayed probe recognition distinguishing between encoding, maintenance and retrieval phases *versus* N-back task confounding all these stages). This approach based on the differentiation of correct-incorrect trials is also problematic since it ideally requires an equal number of correct and incorrect trials in order to obtain identical statistical power for both types of responses, which is often difficult to achieve. Other studies used an inter-individual differences approach by correlating individual performance levels and brain activation profiles, revealing a close relation between better N-back performance and stronger recruitment of a fronto-parietal network in the left hemisphere, higher activity in the amygdala, lower activity in the anterior cingulate cortex, or increased functional connectivity between the posterior and anterior cingulate cortices [16], [17], [18], [19], [20], [21]. However, given the nature of the N-back paradigm, maintenance, encoding and retrieval stages are confounded, and hence it is difficult to determine whether the observed brain-behaviour associations are really due to brain activity during stimulus encoding and maintenance, or mainly stem from activation differences during response decision.

Only a few studies have targeted more specifically brain-behaviour relationships as a function of the different WM stages, by using short duration encoding events and long maintenance intervals, showing that individual differences in activity in the posterior parietal cortex and the left dorsolateral prefrontal cortex during maintenance are positively linked to individual differences in WM performance [4], [22], [23], that activity in dorsolateral prefrontal cortex during retrieval is negatively linked to WM performance [24], or that amygdala activation during maintenance is negatively related to WM performance [14]. These studies, using visuo-spatial memoranda (except for [24]), were optimized for capturing individual differences during the maintenance stage, but much less during the encoding stage since the duration of encoding events was very short as opposed to the duration of the maintenance phase.

This is particularly problematic in the domain of verbal WM where typical tasks, in either experimental (e.g., digit span) or everyday live situations (e.g., maintenance of an unfamiliar phone number), involve multiple, sequentially presented items to be encoded and maintained [3]. Hence, in typical verbal WM situations, encoding is a complex and lengthy process, and individual differences in brain activation profiles during encoding may account for important portions of inter-individual variance in WM performance. The recent focus on attentional accounts of WM further strongly supports the need to consider more closely neural dynamics during encoding in WM tasks [5], [6], [25], [26], [27]. According to these accounts, attentional focalization is one of the main principles of WM and one of the main functions of delay-period activity of the IPS [10], [28]. However, if attentional focalization is a critical mechanism of WM, then this should be even more true for the encoding stage of verbal WM tasks. It is precisely at the encoding stage that task-related attention processes are particularly challenged, as they are needed for efficient processing and encoding of the incoming memoranda; if the attentional focus on memoranda during encoding is diminished or disturbed, further maintenance via attentional refreshing or sustained attention on memoranda will also be compromised.

The present study aimed at furthering our understanding of brain-behaviour interactions in WM, by focusing specifically on verbal WM and on the role of neural activation during the encoding stage which has not been given optimal consideration in past studies although it is a crucial step of WM processing as discussed above. Precisely, according to the attentional account of WM, we hypothesized here that the role of encoding for subsequent WM performance is largely driven by task-related attentional focalization. In order to test this hypothesis directly, we disturbed task-related attentional focalization during encoding by including, in half of the memory lists, unexpected stimuli. These unexpected stimuli were aimed at creating a surprise effect and disturbing task-related attention involved in WM list encoding. As noted earlier, the left IPS has been shown to play a central role for attentional, task-related control during WM tasks [4], [5], [6], [25]. If the left IPS and associated attentional processes determine WM performance during encoding, the level of disruption of left IPS activity in the disturbed encoding condition should predict the subsequent level of WM performance decrement.

The memory lists used in this study were five-item lists, made up of semantically unrelated words or words from a closed and well defined semantic field (i.e., words from positive or negative emotional categories). After a trial containing words from the same semantic field, a new trial with words from the same field was presented; this steady semantic list context was then suddenly interrupted by presenting an unexpected, neutral word, creating a surprise effect aimed at temporarily leading attention away from memory list encoding [6], [29]. The logic behind this procedure was based on semantic habituation experiments where a semantic context is induced and then interrupted by the presentation of a semantically incongruent word; the incongruent stimulus typically leads to a surprise effect, characterized by enhanced brain activity in those areas that support processing of the content of the initial list context [30], [31], [32]. We used here the distinction between emotional versus neutral word categories rather than between other semantic categories (such as tools versus animals) since the impact of emotional words on WM performance, including their occurrence in pure versus mixed WM list contexts, has been extensively explored, allowing us to maximally inform our hypotheses [29], [33]. Precisely, in the present case, given the emotional list context, increased processing in emotional processing areas was expected for the unexpected neutral stimulus, and more specifically in the pons (locus coeruleus) which is not only involved in emotion processing but also in emotion and arousal regulation processes [34], [35], [36], [37]; these processes will intervene when the emotional context changes. When encoding word lists in WM, the surprise effect and the emotion-regulation processes caused by an unexpected stimulus are supposed to temporarily lead task-related attention away from the ongoing encoding of the items of the current memory list, leading to a general decline of encoding performance. One may argue that the unexpected item will increase item distinctiveness and may lead to higher performance levels instead. In order to diminish this possibility, we used closed stimulus sets in this experiment, with all stimuli, expected as well as unexpected ones, being overlearned, which reduces overall item distinctiveness [29], [33], [38]. In order to counterbalance the design, we also included a condition with a majority of neutral words and a single emotional word. No or minimal surprise effects were expected for these trials since a stable semantic context build-up was less likely due to the heterogeneous semantic nature of the neutral words, their only common characteristic being that they were all non-emotional. For all the lists, we used five-item lists since this list length is at or just below WM capacity for word lists in young adults [39], ensuring valid sampling of individual differences in WM performance while avoiding random-level performance in low capacity individuals.

## Methods

### Participants

Twenty-one right-handed native French-speaking young adults (8 male; mean age: 23.71 years; age range: 18–41), with no history of psychological or neurological disorders, were recruited from the university community. The study was approved by the Ethics Committee of the Faculty of Medicine of the University of Liège, and was performed in accordance with the ethical standards described in the Declaration of Helsinki (1964). All participants gave their written informed consent prior to their inclusion in the study.

### Task Description

The memory lists were sampled from a fixed pool of twenty neutral and twenty emotional words. The neutral words came from various semantic categories (*réservoir, assemblage, navette, rondelle, vibration*, [reservoir, assembly, shuttle, slice, vibration]…) while half of the emotional words were from a positively valenced category (*extase, attirance, orgasme, récompense, exploit*, [extasy, attraction, orgasm, reward, achievement] ….) and half from a negatively valence category (*ulcère, agression, divorce, terroriste, inceste*, [ulcer, agression, divorce, terrorist, incest] …). The neutral and emotional words were all 1 to 4 syllable words matched for lexical frequency according to the Brulex database [40] (mean_neutral_: 812.20, range_neutral_: 131–1812; mean_emotional_: 700.75, range_emotional_: 34–2412; t(38)<1, p = .53, η^2^_p_ = .01) as well as for imageability acccording to the database presented in Majerus and D’Argembeau (2011) [29] (mean_neutral_: 4.63, range_neutral_: 3.27–6.32; mean_emotional_: 5.12, range_emotional_: 3.39–7.18; t(38) = 3.07, p = .09, η^2^_p_ = .07). Expectedly, the words differed in terms of emotional valence (mean_neutral_: 4.72, range_neutral_: 3.86–5.36; mean_positive_: 7.91, range_emotional_: 5.73–8.43; mean_negative_: 2.15, range_negative_: 1.73–2.68; F(2,37) = 320.47, p<.001, η^2^_p_ = .95) and arousal (mean_neutral_: 4.79, range_neutral_: 3.86–6.26; mean_positive_: 6.53, range_emotional_: 4.86–7.95; mean_negative_: 7.01, range_negative_: 4.96–7.74 F(2,37) = 20.24, p<.001, η^2^_p_ = .68) [29]. The words were pseudorandomly sampled to construct four types of memory lists: the N and EMO lists were comprised of 5 neutral or five emotional words, respectively; the emotional words for a given list had all the same emotional valence (i.e., all negative or all positive) in order to build up a list-wide emotional-semantic representation; furthermore, words with negative and positive valence were matched for arousal values (t(18) = −1.30, p = .21). Each pure list trial was followed by a disturbing list trial: The EMO list trials were followed by an N_Dist_ list trial where memory lists contained four emotional words, of the same valence as the words of the preceding EMO trial, and one unexpected word sampled from the neutral words, thereby interrupting the emotional context that had been built up and creating a surprise effect; the unexpected word could occur in serial positions 2 to 5, but never in the first serial position, so that the sematic context initiated by the preceding pure list was carried over to the N_Dist_ list, thereby maximizing the surprise effect. In order to counterbalance the design, the same procedure was used for N lists, which were followed by an E_Dist_ list, containing one emotional word occurring in positions 2 to 5 and four neutral words; as already noted, no or minimal surprise effects were expected for these trials due to low between-item semantic predictiveness of the neutral words.

Each list followed the same presentation procedure: during encoding, the stimuli of the memory list appeared in white font in the centre of a black background, in sequential order with a presentation duration of 1250 ms per stimulus; during memory list maintenance, a star in white font appeared in the centre of the screen (variable duration: random Gaussian distribution centred on a mean duration of 7250±2000 ms); at retrieval, a word appeared in the centre of the screen and participants indicated within 3000 ms whether the word matched one of the words of the memory list (by pressing the button under the third finger for ‘yes’ responses and the button under the index for ‘no’ responses). For each of the four list types, there was an equal number of positive and negative probe trials, probing equally all serial positions. Finally, a baseline condition was included, controlling for letter identification and motor response and decision processes; this condition consisted of the presentation of a sequence containing 5 times the same word, followed by a delay interval (a fixation star of variable duration) and a response display showing the same word in upper or lower case; the participants had to decide whether the case was the same as in the target list by pressing the under the third finger or not by pressing the button under the index.

The four WM conditions and the baseline condition were presented in a single session, using an event-related design. There were 30 trials for each STM condition and 18 trials for the baseline condition. The different trials were presented in pseudo-random order, by ensuring that each pure list condition was immediately followed by the corresponding surprise list condition. Before the start of a new trial, an exclamation mark appeared on the centre of the screen during 1000 ms informing the participant about the imminent start of a new trial. The duration of the inter-trial interval was variable (random Gaussian distribution centred on a mean duration of 2000±200 ms) and further varied as a function of the participants’ response times: the probe array disappeared immediately after pressing the response button, followed by the presentation of the next trial. Both response accuracy and response times were collected. Finally, a practice session outside the MR environment, prior to the start of the experiment, familiarized the participants with the specific task requirements and included the administration of ten practice trials.

### MRI Acquisition

The experiments were carried out on a 3 T head-only scanner (Magnetom Allegra, Siemens Medical Solutions, Erlangen, Germany) operated with a standard transmit-receive quadrature head coil. Functional MRI data were acquired using a T_2_*-weighted gradient echo echo-planar imaging (GE-EPI) sequence with the following parameters: TR = 2040 ms, TE = 30 ms, FoV = 192×192 mm^2^, 64×64 matrix, 34 axial slices with 3 mm thickness and 25% inter-slice gap to cover most of the brain. The three initial volumes were discarded to avoid T1 saturation effects. Field maps were generated from a double echo gradient-recalled sequence (TR = 517 ms, TE = 4.92 and 7.38 ms, FoV = 230×230 mm^2^, 64×64 matrix, 34 transverse slices with 3 mm thickness and 25% gap, flip angle = 90°, bandwidth = 260 Hz/pixel) and used to correct echo-planar images for geometric distortion due to field inhomogeneities. A high resolution T_1_-weighted MP-RAGE image was acquired for anatomical reference (TR = 1960 ms, TE = 4.4 ms, TI = 1100 ms, FOV 230×173 mm^2^, matrix size 256×192×176, voxel size 0.9×0.9×0.9 mm^3^). Per session, between 1058 and 1310 functional volumes were obtained. Head movement was minimized by restraining the subject’s head using a vacuum cushion. Stimuli were displayed on a screen positioned at the rear of the scanner, which the subject could comfortably see through a mirror mounted on the standard head coil.

### fMRI Analyses

Data were preprocessed and analyzed using SPM8 software (Wellcome Department of Imaging Neuroscience, http//www.fil.ion.ucl.ac.uk/spm) implemented in MATLAB (Mathworks Inc., Sherbom, MA). EPI time series were corrected for motion and distortion using “Realign and Unwarp” [41] (Andersson et al., 2001) using the generated field map together with the FieldMap toolbox [42] (Hutton et al., 2002) provided in SPM8. A mean realigned functional image was then calculated by averaging all the realigned and unwarped functional scans and the structural T1-image was coregistered to this mean functional image (rigid body transformation optimized to maximize the normalized mutual information between the 2 images). The mapping from subject to MNI space was estimated from the structural image with the “unified segmentation” approach [43]. The warping parameters were then separately applied to the functional and structural images to produce normalized images of resolution 2×2×2 mm^3^ and 1×1×1 mm^3^ respectively. The scans were screened for motion artefacts and time series with motion peaks exceeding 3 mm (translation) or 3° (rotation) were discarded. Finally the warped functional images were spatially smoothed with a Gaussian kernel of 8 mm full-width at half maximum (FWHM).

For each subject, brain responses were estimated at each voxel, using a general linear model with epoch and event-related regressors. We assessed transient activation events, using distinct regressors for the encoding, maintenance and retrieval events, as a function of WM condition; for the encoding event, regressors modelled each target stimulus separately (the unexpected stimulus and the same-position stimulus from the previous pure list). The maintenance regressor covered the duration of the entire duration phase until the onset of the retrieval probe display. The retrieval regressor covered the duration of the retrieval probe display until the response of the participant. The variable duration of the maintenance regressor ensured minimal auto-correlation between the early maintenance and the other regressors [44], [45], [46], [47]. The baseline condition was modelled implicitly meaning that any activation reported in this study is activation controlled for baseline activation. Boxcar functions representative for each regressor were convolved with the canonical hemodynamic response. The design matrix also included the realignment parameters to account for any residual movement-related effect. A high pass filter was implemented using a cut-off period of 128s in order to remove the low frequency drifts from the time series. Serial autocorrelations were estimated with a restricted maximum likelihood algorithm with an autoregressive model of order 1 (+ white noise).

One linear contrast for each of the twelve cells resulting from the crossing of the four conditions and the three WM events were defined. The resulting set of voxel values constituted a map of t statistics [SPM{T}]. These contrast images were then smoothed again (6-mm FWHM Gaussian kernel) in order to reduce remaining noise due to inter-subject differences in anatomical variability in the individual contrast images. Smoothing by 8 mm (at the first level) then by 6 mm leads to a single equivalent smoothing kernel of 10 mm (as 10^2^ = 8^2^+6^2^), a common value for multiple subject analysis. Given the linear nature of the general linear model used here, smoothing can be applied at any stage of processing. The use of a two-step smoothing procedure was justified by the fact that we used low levels of smoothing for the estimation of the data at the single-subject level; these data were used for the extraction of individual volumes of interest for the psychophysiological interaction analyses (see below). The additional smoothing by 6 mm then allowed us to attain the more common levels of smoothing for group-level analyses. The contrast images were then entered in second-level, random effect analyses. A first analysis used null conjunction analyses to determine common activations across all four conditions, as a function of WM phase. A second analysis assessed differential effects between the different conditions, as a function of WM phase. A third analysis assessed brain-behaviour correlations, by regressing behavioural results (response accuracy) on contrast images (see Results section for further details). As a rule, statistical inferences were performed at the voxel level at *p*<0.05 corrected for multiple comparisons across the entire brain volume using Random Field Theory [48]. For regions of interest not significant at this level, a small volume correction [49] was applied on a 10-mm radius sphere around coordinates-of-interest published in previous studies (see below).

An additional model assessed functional connectivity patterns between the N and N_Dist_ conditions during encoding using psychophysiological interaction analysis. This analysis determined whether the correlations between activity in the seed region (left posterior IPS; see results) and other brain regions differed in the N_Dist_ and N trials [50], [51], and whether any differential functional connectivity patterns between these two conditions were related to WM performance differences in these two conditions. A new linear model was constructed for each subject, using three regressors (plus the realignment parameters). One regressor represented the N_Dist_ condition of interest relative to the N condition. The second regressor was the activity in the seed region extracted for each subject. The third regressor represented the interaction of interest between the first (psychological) and second (physiological) regressors. Significant contrasts for this psychophysiological regressor indicated a change in the regression coefficients between any reported brain area and the reference region, in the N_Dist_ condition relative to the N condition. After smoothing (6-mm FWHM Gaussian kernel), these contrast images were then entered in a second-level (random effects) analysis and regressed on differential WM performance measures. One-sample t-test assessed the significance of the correlation between WM performance and functional connectivity patterns.

### A Priori Locations of Interest

Regions of interest included the bilateral IPS as well as bilateral ventrolateral and dorsolateral prefrontal consistently activated in verbal WM tasks as discussed in the Introduction section. Furthermore, given the manipulation of emotional semantic content for creating distraction within the WM lists, regions of interest also included regions known to be sensitive to emotional semantic content and regulation.

WM: SMA [−12, 32, 32] [5], dorsolateral prefrontal cortex [−50, 2, 40; −42, 30, 30; 50, 26, 34] [5], [7], [44], [45], [46], [52]; ventrolateral prefrontal cortex [−48, 19, 7; −48, 44, 2; −58, 12, 14; 52, 16, 2] [5], [45], [52], [53]; anterior IPS [−40, −36, 40; 42, −38, 44] [5], [25]; posterior IPS [−26, −62, 46] [6]; precentral gyrus [57, −2, 42] [5], [53];

Emotion semantics and regulation: anterior cingulate [0, 24, −6] [36], [52]; pons [−9, −21, −18] [34]; angular gyrus [−30, −58, 42] [35].

## Results

### Behavioural

**Figure 1:**
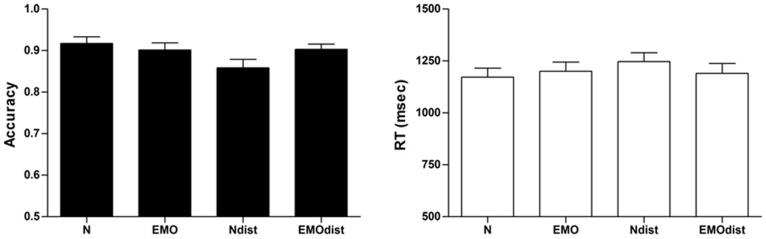
Accuracy and response times for behavioral performance in the working memory task, as a function of stimulus condition (N = pure neutral stimuli; EMO = pure emotional stimuli; Ndist = list with emotional standard stimuli and one unexpected neutral stimulus; EMOdist = list with neutral standard stimuli and one unexpected emotional stimulus).

A first ANOVA, with semantic category and list type as repeated measures, assessed whether there was a reliable effect of list condition on WM accuracy. A main effect of list type, F(1, 20) = 5.05, p<.05, η^2^ = .20, and a significant list type by semantic category interaction, F(1, 20) = 6.00, p<.01, η^2^ = .23, were observed; the main effect of semantic category was not significant, F(1, 20)<1.00, p = .36, η^2^ = .04. As expected, Bonferroni post-hoc comparisons showed that performance decreased specifically for the emotional lists containing one unexpected neutral word, relative to the pure neutral list (p<.01); no other comparisons were significant (see Figure 1). We also had predicted that this effect was due to overall lower encoding performance of the whole memory list rather than a specific item This was explored by a second ANOVA comparing recognition performance for unexpected and standard words in lists containing one unexpected word, with expectedness status and semantic category as repeated measures: we observed no main effect of expectedness status, F(1, 20)<1.00, p = .95, η^2^ = .01, no main effect of semantic category, F(1, 20)<1.00, p = .37, η^2^ = .04, but a significant stimulus status by semantic category interaction, F(1, 20) = 7.60, p<.05, η^2^ = .28; this interaction was characterized by lower performance for unexpected neutral words (mean = 83, SE = .02) and same-list standard emotional words (mean = .85, SE = .02), relative to unexpected emotional words (mean = .88, SE = .02) and same-list standard neutral words (mean = .90, SE = .02); post-hoc comparisons showed a significant difference for unexpected neutral versus different-list standard neutral words, p<.05. Also, there was no evidence for any intervention of distinctiveness, since the list with the theoretically highest potential for distinctiveness, the neutral lists containing one emotional word, did not lead to higher performance relative to the neutral pure lists; this was also confirmed by the second ANOVA directly comparing the unexpected emotional word versus the same-list standard words. Next we explored response times. Again, list type and semantic category significantly interacted, F(1, 20) = 12.19, p<.001, η^2^ = .38; the main effects for list type, F(1, 20) = 2.61, p = .12, η^2^ = .12, and semantic category, F(1, 20) = 1.44, p = .24, η^2^ = .07, were not significant. Bonferroni post-hoc comparisons showed significantly slower responses for the emotional lists containing one unexpected neutral word, relative to the pure neutral list condition (p<.01) (see Figure 1). As for response accuracy, we also performed a second ANOVA comparing unexpected words and same-list standard words. We observed no main effect of expectedness status, F(1, 20) = 2.80, p = .11, η^2^ = .12, no main effect of semantic category, F(1, 20) = 1.93, p = .18, η^2^ = .09, and a marginally significant expectedness status by semantic category interaction, F(1, 20) = 3.70, p = .07, η^2^ = .16; this interaction was characterized by longer reaction times for standard emotional words (mean = 1274, SE = 48) relative to same-list unexpected neutral words (mean = 1185 SE = 40, p<01), or relative to standard neutral words (mean = 1194, SE = 48, p<.01) and same-list unexpected emotional words (mean = 1185 SE = 47, p<.05). In sum, the behavioural results led to the expected pattern, with altered performance for lists containing an unexpected emotional-semantic event, and this specifically for unexpected neutral stimuli in the context of emotional lists where emotional-semantic context expectations were the highest; this effect concerned both the target unexpected word, as shown by significantly reduced response accuracy, and the same-list standard emotional words as shown by lower response accuracy and significantly reduced response times. As noted, a list effect was not expected for neutral lists containing one emotional word since the neutral words stemmed from different semantic contexts, and hence these lists did not induce a strong semantic list-wide expectation.

### Neuroimaging

#### Impact of list type on WM activation patterns

First, we checked for expected fronto-parietal activation patterns across the four conditions, using null conjunction analyses over the four WM conditions. For the encoding and retrieval phases, wide-spread fronto-parieto-cerebellar activity was observed (see Table 1); note that common activation in the parietal target area, the left IPS, was very small and restricted to 6 voxels in the most posterior part of the IPS. The maintenance stage did not elicit significant specific activation, in line with previous studies using WM paradigms with encoding events of relatively long duration and maintenance events of variable duration [5], [45].

**Table 1:** Common transient activation peaks (null conjunction) for the four experimental conditions, as a function of encoding, maintenance and retrieval.

Anatomical region	No. voxels	Left/right	x	y	z	BA area	SPM {Z}-value
**Encoding**							
SMA/ACC	1240	B	−4	14	48	6/32	7.07
Middle frontal gyrus	1010	L	−48	2	48	6	5.75
Middle frontal gyrus	48	R	60	4	42	6	4.00 ?
Superior frontal gyrus	338	R	38	48	30	9	4.82
Inferior frontal gyrus/Insula	456	L	−40	16	0	47/13	6.13
Inferior frontal gyrus/Insula	253	R	38	18	0	47/13	5.25
Intraparietal sulcus (post)	6	L	−28	−58	38	40	3.32*
Intraparietal sulcus (ant)	85	R	44	−32	38	40	4.38*
Superior temporal gyrus	392	L	−56	−44	6	22	6.89
Middle temporal gyrus	280	L	−62	−26	−2	21	6.93
Globus pallidum	247	L	−18	−2	0		5.29
Cerebellum	445	L	−34	−88	−18		7.82
Cerebellum	490	L	−24	−96	−6		6.04
Cerebellum	172	L	−40	−42	−22		4.81
Cerebellum	1353	R	34	−66	−30	CrI	5.35
**Maintenance**							
no voxel above threshold							
**Retrieval**							
ACC	506	B	−2	2	38	24	6.04
Medial frontal gyrus	508	R	8	32	20	9	6.83
Middle frontal gyrus	387	L	−36	58	2	10	4.54
Middle frontal gyrus	461	R	22	54	−10	10	7.28
Inferior frontal gyrus	502	L	−36	14	−10	47	6.16
Inferior frontal gyrus	515	R	34	16	−14	47	>7.80
Insula	486	L	−42	−4	10	13	>7.80
Insula	485	R	42	0	8	13	5.96
PCC	450	R	12	−66	8	30	6.02
Postcentral gyrus	503	L	−54	−22	44	2	>7.80
Postcentral gyrus	511	R	56	−14	20	43	6.56
Supramarginal gyrus	515	L	−52	−22	18	40	>7.80
Intraparietal sulcus (anterior)	183	L	−48	−32	56	40	>7.80
Intraparietal sulcus (posterior)	67	L	−36	−62	44	40/7	3.96
Intraparietal sulcus (anterior)	19	R	46	−48	40	7/40	3.61
Intraparietal sulcus (posterior)	71	R	38	−58	40	7/40	3.80
Superior temporal gyrus	492	R	56	−46	4	22	5.72
Inferior temporal gyrus	502	L	−44	−70	0	37	5.96
Precuneus	389	R	6	−70	38	7	5.27
Occipital gyrus	504	L	−34	−88	−12	18	>7.80
Occipital gyrus	482	R	40	−82	−12	18	>7.80
Thalamus (mammilary body)	515	L	−12	−18	4		>7.80
Thalamus (mammilary body)	502	R	14	−12	10		7.21
Cerebellum	515	L	−34	−54	−30	VI	7.11
Cerebellum	513	L	−36	−72	−18		>7.80
	513	R	30	−54	−24		>7.80
	497	R	44	−74	−14		>7.80

If not otherwise stated, all regions are significant at p<.05, corrected for whole brain volume.

* p<.05, small volume corrections

**Figure 2:**
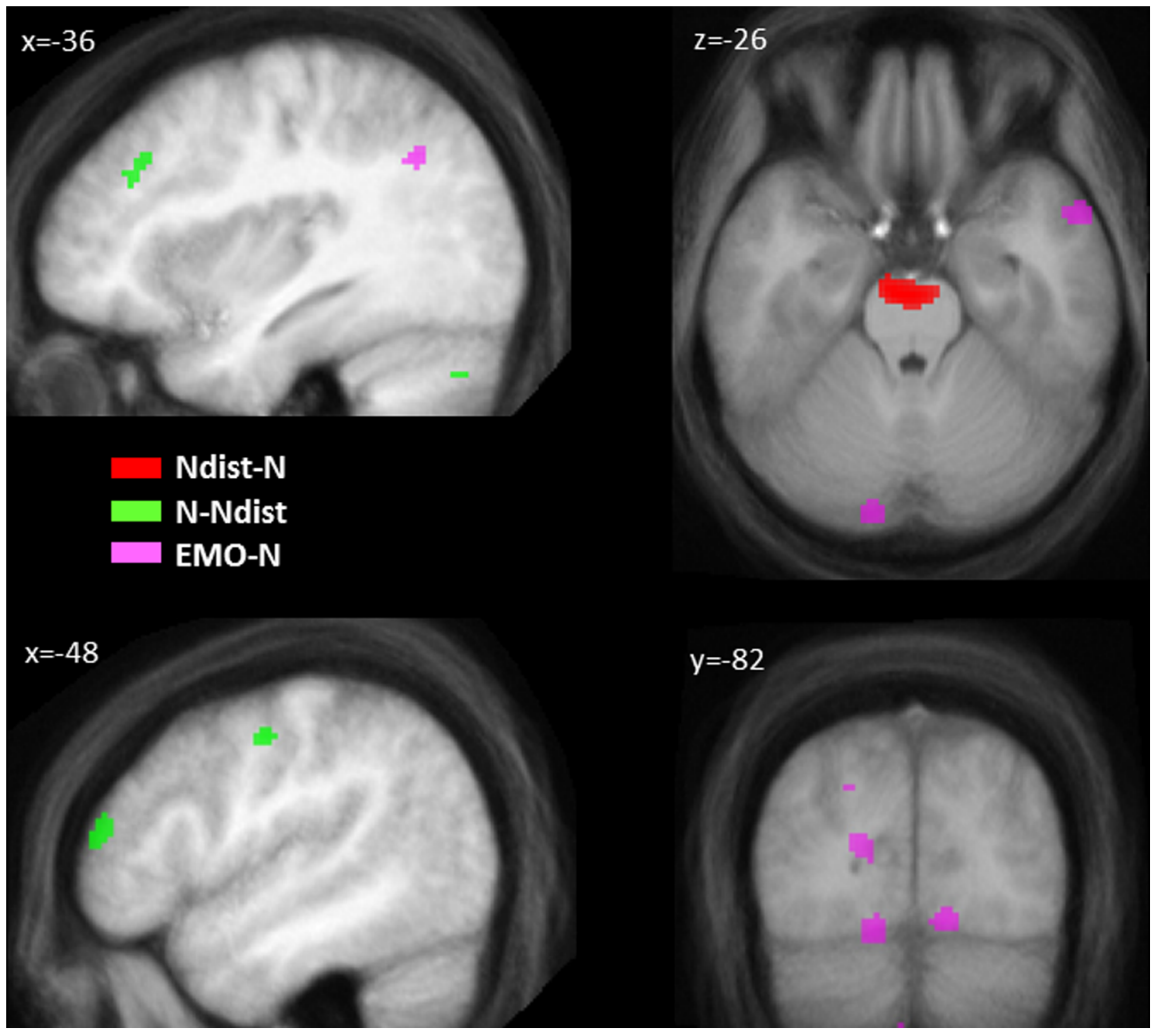
Regions showing significant differential activation during encoding as a function of WM condition, with a display threshold of p<.001, uncorrected (N = pure neutral stimuli; EMO = pure emotional stimuli; Ndist = list with emotional standard stimuli and one unexpected neutral stimulus).

Next, we determined the impact of list type on WM encoding activity, by focussing specifically on the lists containing one unexpected neutral word and four emotional words, for which a surprise effect was expected and had been confirmed by the behavioural results. The unexpected neutral word, as compared to same position items from neutral pure lists, was associated with activation in the anterior part of the upper brainstem, in the area of the locus coeruleus bilaterally (see Table 2 and Figure 2). This region of the upper brainstem is known to be associated with arousal and arousal regulation [34], [37], [54]. Hence the participants reacted to the unexpected neutral word in an emotional list context with a surprise response characterized by increased arousal and emotion regulation. Also, as expected, emotional words led to increased activation in areas associated with emotional semantic processing such as the bilateral lingual gyrus and the left angular gyrus, relative to neutral words in a pure list context (see Table 2 and Figure 2). Finally, encoding of neutral words in a pure list context, relative to unexpected neutral words in an emotional list context, was associated with increased activation in dorsal and ventral lateral prefrontal cortex as well as cerebellar components of the WM network identified in the earlier conjunction analyses, suggesting more efficient recruitment of prefrontal and cerebellar parts of the WM network when no unexpected stimulus occurs (see Table 2). No condition-specific activation patterns were observed for maintenance and retrieval stages, except for increased bilateral anterior cingulate cortex activation when retrieving words from pure neutral lists, relative to words from surprise lists or from pure emotional lists.

**Table 2:** Differential transient activation peaks for between-condition comparisons, as a function of encoding, maintenance and retrieval.

Anatomical region	No. voxels	Left/right	x	y	z	BA area	SPM {Z}-value
**Encoding**							
**N_Dist_-N**							
Pons (anterior)	37	B	−4	−18	−26		4.47*
**N-N_Dist_**							
Middle frontal gyrus	22	L	−38	34	28	9	3.24*
Middle frontal gyrus	2	L	−46	−6	44	4	3.60*
Inferior frontal gyrus	5	L	−48	44	12	46	3.37*
Cerebellum	946	L	−24	−70	−40		4.21
Cerebellum		L	−4	−64	−40		3.97
Cerebellum		L	−22	−52	−42		3.95
Cerebellum	563	R	24	−64	−46		4.45
		R	16	−48	−42		4.09
**EMO-N**							
Angular gyrus	18	L	−36	−60	36	39	3.66*
Lingual gyrus	410	L	−4	−96	−6	18	4.95
Lingual gyrus	310	R	12	−88	−12	18	4.59
Cerebellum	413	L	−10	−90	−18		5.89
**N-EMO_,_ EMO_Dist_-N**							
no voxel above threshold							
**N-EMO_Dist_**							
no voxel above threshold							
**Maintenance**							
no voxel above threshold for any contrast							
**Retrieval**							
**N_Dist_-N, EMO-N_Dist,_ EMO_Dist_-N_,_ N-EMO_Dist_**							
no voxel above threshold							
**N-N_Dist_**							
ACC	76	L	−8	28	−2	24	3.38*
ACC	25	R	4	24	0	24	3.37*
**N-EMO**							
SMA/ACC	134	L	−14	30	24	9	3.99 *

If not otherwise stated, all regions are significant at p<.05, corrected for whole brain volume.

* p<.05, small volume corrections

N = pure neutral stimuli; EMO = pure emotional stimuli; Ndist = list with unexpected neutral stimulus and emotional standard stimuli

### Neural Dynamics Predicting WM Performance

**Figure 3:**
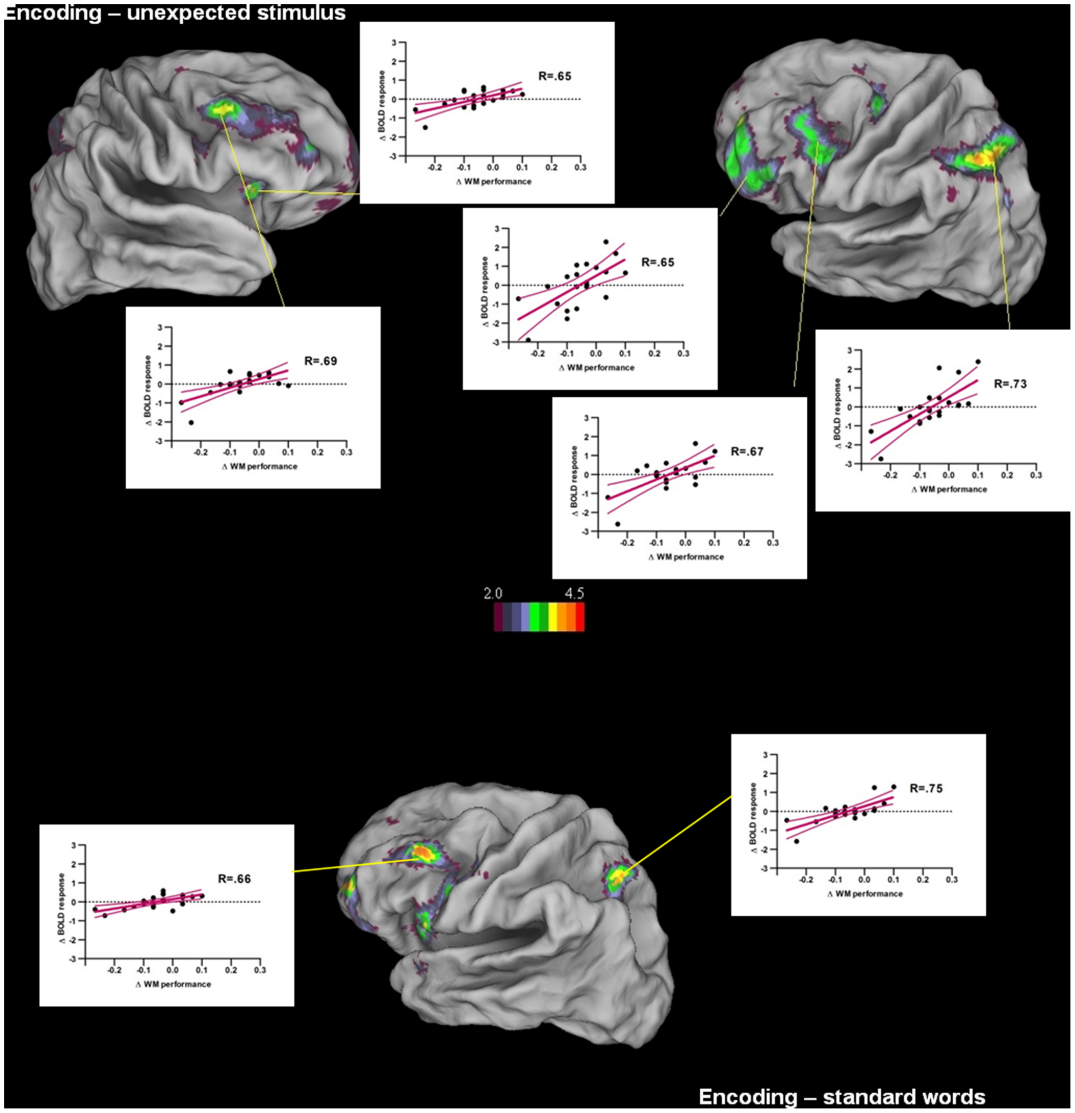
Differential activation foci for the unexpected neutral minus pure neutral conditions showing significant correlation with differential behavioural performance for the two same conditions, with a display threshold of 2≤ Z≤4.5, p<.001, for distractor stimulus-specific encoding (top half of figure) and post-distractor standard word-specific encoding (bottom half of figure) events. The scatterplots indicate differential working memory performance (x-axis) as a function of differential estimated BOLD response (y-axis) for each region, with regression lines, 95% confidence bands and the value of r.

After having demonstrated a reliable impact of the unexpected neutral stimulus on encoding-related neural activity and behavioural results, we determined the relationship between them by regressing differential WM performance levels for lists containing an unexpected neutral word relative to the pure neutral word lists on differential brain activation patterns for the same two conditions. For stimulus-specific activity during the encoding stage, we observed a strong positive correlation: participants maintaining stable WM performance for lists with an unexpected neutral word were also those who maintained stable activation patterns in the fronto-parieto-cerebellar WM network when encoding the unexpected neutral word (see Table 3 and Figure 3). Importantly, the strongest brain-behaviour correlation was observed in the anterior and posterior parts of the left IPS; this region had also shown the least robust activation in the previous conjunction analyses assessing common activation patterns during encoding across the four WM conditions. These results suggest that, as predicted, the left IPS activation is more directly related to individual differences in maintaining a task-related attentional focus in the presence of unexpected list events and is critical for ensuring accurate task-related list encoding which predicts subsequent WM performance. On the other hand, differential activation levels of the left IPS during maintenance and retrieval do not appear to be predictive of WM performance: during maintenance and retrieval, no significant brain-behaviour correlations were observed. Bayesian estimation confirmed that the role of differential IPS activity during maintenance and retrieval stages on final WM performance outcome is negligible: there was a less than 5% chance for the IPS of being involved, and this for even a very small effect size of.20. Furthermore, we determined whether the brain-behavior correlation observed during encoding was specific to the moment where the unexpected stimulus occurs, or whether it was a related to a broader list-context effect, as suggested by the behavioural results showing an impact of stimulus unexpectedness not only on recognition of the unexpected stimulus but also of same-list standard stimuli. We separated the encoding phase in before-unexpected-stimulus and after-unexpected-stimulus epochs and ran the same brain-behavior correlation analyses as before on these epochs: there was a significant correlation for the after-unexpected-stimulus epoch in the left posterior IPS, as well as in the left dorso-lateral prefrontal cortex (see Table 3). Also, as expected, there were no significant brain-behavior correlations when focussing on the before-unexpected-stimulus epoch. In other words, participants who maintained stable WM performance in the context of an unexpected stimulus were also more likely to maintain stable IPS activation, and this both during and after the encoding of the unexpected stimulus, relative to encoding of same position events in pure neutral lists.

**Table 3:** Differential activation foci for the unexpected neutral minus pure neutral conditions showing significant correlation with differential behavioral performance for the two same conditions, as a function of STM phase.

Anatomical region	No. voxels	Left/right	x	y	z	BA area	SPM {Z}-value	95% confidenceinterval for r
**Encoding – Unexpected stimulus**								
Middle frontal gyrus	137	R	54	28	38	9	3.61*	.36–.86
Middle frontal gyrus	3	L	−38	22	28	9	3.19*	.31–.85
Inferior frontal gyrus	12	L	−52	36	0	47	3.32*	.33–.85
Intraparietal sulcus (posterior)	140	L	−28	−58	48	40/7	3.78*	.44–.88
Intraparietal sulcus (anterior)	18	L	−44	−44	36	40	3.37*	.35–.86
**Encoding – Standard words**								
Middle frontal gyrus	7	L	−42	30	30	9	3.26*	.32–.85
Intraparietal sulcus (posterior)	81	L	−24	−66	54	40/7	3.91*	.48–.89
**Transient - Maintenance**								
no voxel above threshold								
**Transient - Retrieval**								
no voxel above threshold								

* p<.05, small volume corrections

### Functional Connectivity – Psychophysiological Interaction

**Figure 4:**
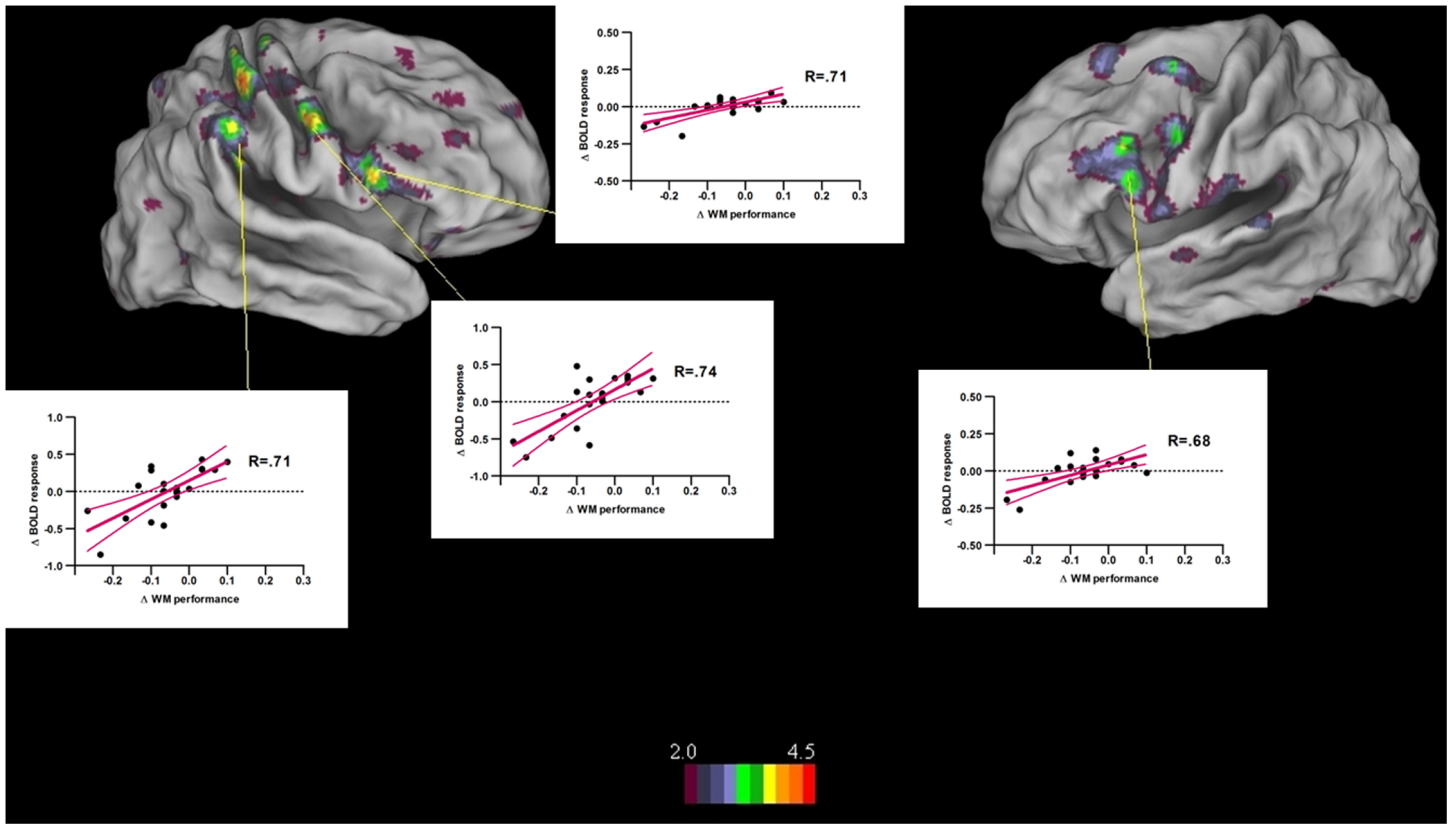
Differential functional connectivity patterns for the unexpected neutral versus pure neutral conditions showing significant correlation with differential behavioral performance for the two same conditions, with a display threshold of 2≤ Z≤4.5, p<.001, for stimulus-specific encoding events. The scatterplots indicate differential working memory performance (x-axis) as a function of differential functional connectivity strength (y-axis) for each region with regression lines, 95% confidence bands, and the value of r.

Task-related attentional disruption created by the unexpected neutral stimulus should not only impact activation levels of the left IPS supporting task-related attention, but will also likely lead to a disruption of the coupling between parietal and prefrontal components of the WM network during encoding. Discoupling of parietal and prefrontal components is supported by the fact that encoding of the unexpected neutral words led to generally diminished activation levels in the lateral prefrontal cortex but not the left IPS. To test this possibility and its impact on behavioural performance, we determined functional connectivity patterns between parietal and prefrontal sites via psychophysiological interaction analyses. The left posterior IPS [−28, −58, 48] was taken as a seed region since this area had shown the strongest correlation with behavioural performance in the preceding analyses. We observed that functional connectivity was decreased when the unexpected neutral stimulus had to be encoded, and this specifically between the left IPS and bilateral ventrolateral prefrontal cortex (see Table 4 and Figure 4). Importantly, individual differences in differential functional connectivity patterns predicted WM performance: WM performance for lists containing an unexpected neutral word was most preserved in those participants who showed the smallest decrease in functional connectivity strength between the left IPS, premotor and ventrolateral prefrontal cortex when encoding the unexpected neutral stimulus.

**Table 4:** Functional connectivity patterns (psychophysiological analysis) for the left IPS as seed region, during unexpected neutral versus pure neutral stimulus encoding.

Anatomical region	No. voxels	Left/right	x	y	z	BA area	SPM {Z}-value	95% confidence interval for r
**Transient - Encoding** ** Increased connectivity**								
no voxel above threshold								
** Decreased connectivity**								
Inferior frontal gyrus	1	L	−56	18	14	44	3.18*	/
Inferior frontal gyrus	21	R	60	18	6	45	3.58*	/
*** Correlation with WM performance***								
Inferior frontal gyrus	8	L	−62	26	10	44/45	3.39**	.35–.86
Inferior frontal gyrus	13	R	60	38	2	45	3.60**	.40–.87
Precentral gyrus	54	R	52	0	36	6	3.82*	.45–.89

* p<.05, small volume corrections;

** p<.001, uncorrected

## General Discussion

The present study highlights the importance of neural dynamics during the encoding stage for predicting inter-individual differences in verbal WM performance, by showing that, in the context of task-related attentional disruption during WM encoding, encoding-related activity of the left anterior and posterior IPS significantly correlates with subsequent performance during verbal WM retrieval. Furthermore, inter-individual differences in verbal WM performance were not only predicted by activation patterns in the left IPS during occurrence of the attention-disrupting unexpected stimulus, but also by left IPS activation patterns for encoding of subsequent standard stimuli. Finally, we demonstrate that encoding-related functional connectivity strength between the left posterior IPS and ventrolateral prefrontal cortex further predicts inter-individual variability in verbal WM performance.

### Implications for the Study of Neural Dynamics Supporting Inter-individual Differences in Verbal WM

The present study provides new insights into the brain dynamics predicting WM performance, and this specifically for verbal WM paradigms which typically are characterized by long-duration and attention demanding encoding events. The results of the present study contrast with previous studies, by showing that posterior parietal cortex activation is associated with WM performance more specifically due to the nature of transient activation patterns during encoding, but not during maintenance in the delay period. The relevance of brain dynamics during the encoding stage for subsequent WM performance has been neglected as so far by studies exploring WM brain-behaviour interactions [4], [15], [22], [23]. A study using a verbal WM paradigm closer to the one used in the present also observed no correlation with WM performance for delay-specific activation in the posterior parietal cortex [24]. Thus, for verbal WM paradigms with long encoding durations, it is transient anterior and posterior parietal activation during encoding which appears to be critical for accurate WM performance. At the same time, we should note that the results of our study do not imply that delay-specific activity of the IPS plays no role at all for the prediction of WM performance; rather, given the design used here relating behavioural performance to differential activity levels between two WM conditions (neutral versus emotional-semantic surprise conditions), our study suggests at a minimum that individual variations in differential activation levels between the two conditions during the delay period do not predict variations in subsequent differential WM performance levels.

At the same time, like in previous brain-behaviour correlation studies for both visual and verbal WM domains, the role of dorsolateral prefrontal cortex in predicting WM performance is also confirmed, by showing that encoding-related activity in the dorsolateral prefrontal cortex is an additional important predictor of WM performance, [15], [22]. The dorsolateral prefrontal cortex may play a more general role by protecting WM content against interference from irrelevant internal or external information (such as scanner noise, internal thoughts, etc…) [13]. This is clearly consistent with the design of the present study, where the internal thoughts and reactions elicited by the unexpected stimulus during encoding had to be suppressed in order to maintain stable WM performance. It is interesting to note that Todd and Marois (2005) [23] obtained dissociations between the respective roles of the parietal cortex and the dorsolateral prefrontal cortex in WM performance, by showing that activity in the posterior parietal cortex correlated with k, an estimate of individual WM capacity and amounting to about 4 items (for the type of visual WM task used in that study), while dorsolateral prefrontal involvement in WM performance was indicative of a WM subcapacity, by levelling off at about 2 items. This supports recent assumptions that parietal and prefrontal contributions to WM performance are due to the intervention of distinct mechanisms.

A further novel finding of this study is the demonstration of functional connectivity patterns between posterior parietal cortex and ventrolateral prefrontal cortex during encoding as being reliable determinants of inter-individual differences in WM performance. One previous study assessed functional connectivity patterns and their relationship to WM performance, by focusing on anterior and posterior cingulate cortices [19]. Our results show that the posterior parietal cortex and the ventrolateral prefrontal cortex are functionally connected during encoding of memoranda, and that the level of disruption of this functional connectivity as a result of unexpected stimuli occurring during encoding is indicative of subsequent WM performance. These results also support a study that explored functional networks via structural equation modelling, and which showed that participants with the highest performance on an n-back task used a left-sided fronto-parietal network including the left inferior parietal cortex and Broca’s area, close to the left ventrolateral prefrontal cortex involvement observed in the present study [17].

One limitation of the present study exploring inter-individual differences at behavioural and neural levels of WM is its relatively low sample size, urging for caution as regards the generalizability of the present results to the general population. At the same time, despite the low sample size and the resulting large confidence intervals surrounding the brain-behaviour correlations, the lower bound population estimates of the correlations were clearly different from null correlations (all 95% lower bound estimates of R were higher than .30), suggesting that the brain-behavior correlations observed here show nevertheless a satisfactory reliability. We however need to be cautious about the null results observed for brain-behavior correlations during the maintenance stage, which could be caused by the low sample size.

### Inter-individual Differences in Neural Dynamics during Verbal WM: a Signature of Inter-individual Differences in Task-related Attentional Control?

Pessoa et al. (2002) (page 984) [15] wrote: “We attribute the coupling between brain activity and performance to trial-to-trial fluctuations in attention, such that variability in the subjects’ attention leads to variability in neuronal responses, which, in turn, cause variability in performance.” We provide in this study some direct evidence for this assertion by showing that resistance to attentional distraction created by the occurrence of an unexpected stimulus event during encoding is strongly associated t with both stable WM performance and stable activation in the posterior parietal cortex. The present study further suggests that this ability to keep a task-related attentional focus during WM tasks is related to the parietal cortex, since left posterior and anterior IPS activation, previously associated with the dorsal, task-related attention network [4], [6], [23], predicted WM performance during encoding, when the unexpected stimulus occurred, and right after the unexpected stimulus. The present data suggest that the left IPS may play a central role in the maintenance of task-related attentional control in the context of the occurrence of unexpected stimuli during encoding. This is further supported by the fact that a brain-behavior correlation was observed for IPS activity not only during occurrence of the unexpected stimulus, but also for subsequent standard word encoding : this shows that IPS activity does not just reflect detection of the unexpected stimulus, but exerts a wider attentional control function over list context. This argument is also valid for the involvement of the dorsolateral prefrontal cortex, with activity both at and after the unexpected stimulus correlating with WM performance. The function of this region may be to protect encoded information against interference from internal thoughts created by the unexpected stimulus as suggested by Postle (2005), as already noted [13]. At the same time, task-related attention mechanisms in the IPS need to be coupled with prefrontal cortex activation, as suggested by our functional connectivity analyses showing that WM performance is predicted by functional connectivity strength between the left IPS and ventrolateral prefrontal cortex involved in rehearsal [11].

More generally, our results are in line with a growing literature highlighting the importance of attentional control mechanisms for WM performance [2], [9], [26]. Lewis-Peacock and Postle [28] showed that supposedly delay-specific and maintenance-specific activation during WM tasks may in fact be better described as reflecting reactivation or sustained activation of the focus of task-related attention. As we have seen, especially the posterior parietal cortex has been associated with attentional control processes during WM [4], [5], [6], [10], [25], [45], [46]. The posterior parietal cortex has also been identified as supporting an attentional selection function, by selecting to-be-maintained items and ignoring other items in WM tasks [4], [6], [25], [55]. The present study provides new support for the attentional account of WM, by associating WM brain dynamics during and after task-related attentional disruption to inter-individual differences in subsequent WM performance.
